# A pilot study of a practice management training module for medical residents

**DOI:** 10.1186/1472-6920-14-107

**Published:** 2014-05-24

**Authors:** Lizanne Berkenbosch, Arno M M Muijtjens, Luc J I Zimmermann, Ide C Heyligers, Albert J J A Scherpbier, Jamiu O Busari

**Affiliations:** 1 School of Health Professions Education, Faculty of Health, Medicine and Life Sciences, Maastricht University, P.O. Box 616, 6200, MD, Maastricht, the Netherlands; 2 Educational Development & Research, Faculty of Health, Medicine and Life Sciences, Maastricht University, P.O. Box 616, 6200, MD, Maastricht, the Netherlands; 3 Department of Paediatrics, Maastricht University Centre Maastricht, P.O. Box 5800, 6202, AZ, Maastricht, the Netherlands; 4 Department of Orthopaedic Surgery, Atrium Medical Center, Henri Dunantstraat 5, 6401CX Heerlen, The Netherlands; 5 Maastricht University medical Centre, P.O. Box 5800, 6202, AZ, Maastricht, the Netherlands; 6 Department of Educational Research & Development, Faculty of Health, Medicine and Life Sciences, Maastricht University, P.O. Box 616, 6200, MD, Maastricht, the Netherlands

**Keywords:** Medical residents, Management training, Competency, Postgraduate curriculum

## Abstract

**Background:**

In 2005 a competency based curriculum was introduced in the Dutch postgraduate medical training programs. While the manager’s role is one of the seven key competencies, there is still no formal management course in most postgraduate curricula. Based on a needs assessment we conducted, several themes were identified as important for a possible management training program. We present the results of the pilot training we performed to investigate two of these themes.

**Methods:**

The topics “knowledge of the healthcare system” and “time management” were developed from the list of suggested management training themes. Fourteen residents participated in the training and twenty-four residents served as control. The training consisted of two sessions of four hours with a homework assignment in between. 50 True/false-questions were given as pre- and post-test to both the test and control groups to assess the level of acquired knowledge among the test group as well as the impact of the intervention. We also performed a qualitative evaluation using evaluation forms and in-depth interviews.

**Results:**

All fourteen residents completed the training. Six residents in the control group were lost to follow up. The pre- and post-test showed improvement among the participating residents in comparison to the residents from the control group, but this improvement was not significant. The qualitative assessment showed that all residents evaluated the training positively and experienced it as a useful addition to their training in becoming a medical specialist.

**Conclusion:**

Our training was evaluated positively and considered to be valuable. This study supports the need for mandatory medical management training as part of the postgraduate medical curriculum. Our training could be an example of how to teach two important themes in the broad area of medical management education.

## Background

Today’s doctors differ from their predecessors and work in a different health care environment. The physician of a century ago for example, was usually male and always available. He took decisions about his patients alone and rarely was there joint deliberation with colleagues within his own or other specialties. Nowadays, physicians practice their profession as part of a team across multiple disciplines and decisions are made within the context of multidisciplinary guidelines and institutional frames of reference. In addition, more doctors are working part-time, are on the payroll of hospitals and women are increasingly taking on the professional role as physicians [1,2]. In the Netherlands, as in some other countries, the healthcare system and parts of the social security system are undergoing a transformation, moving from a framework of price regulation to one of regulated market forces [3-5]. Due to this transition, physicians are to a greater extent than before, compelled to take part in negotiations with insurers and hospital management [2]. The role for physicians as medical managers is in this context of increasing importance. On the first of January 2005 a new competency based curriculum was introduced in the postgraduate medical training programs of Dutch medical residents [6]. The new curriculum was based on seven competencies that residents were expected to possess upon graduation (i.e. medical expert, communicator, collaborator, manager, health advocate, professional and scholar) and is derived from the CanMEDS framework from Canada [7]. Although the CanMEDs competencies were introduced in 2005 and the manager’s role is gaining increased importance, there is still no formal national curriculum in the Netherlands that teaches this role.

Hence, we decided to investigate if there was a need for management training among residents in the Netherlands and if so, what such training program should look like. We started by performing a literature review to find out what already had been written on the topic [8] and we performed a survey and needs assessment among Dutch medical residents and specialists on the perceived management competencies of junior doctors and their training needs [9-11]. The most important results of these projects are summarized in Table 1.

**Table 1:** Overview of previously performed research by the authors

**Research project**	**Most important findings**
Literature review [8]	• 40 articles on medical management were found
	• 24 articles described management curricula
	• The curricula differed in timing, length, content and teachers, but were all evaluated positively
	• Topics most taught: financial concepts, management concepts, quality assurance, legal issues, personnel issues and organizational skills/time management
Perceived competencies by residents [10]	• Neutral perceptions on: negotiating personal ambitions, possessing adequate leadership skills, knowledge of the legal aspects of healthcare and knowing how to deal with medical errors
	• Inadequate perceptions on: contract negotiating skills and knowledge of how the healthcare system and specialists departments are financed and organized
Needs assessment among residents [11]	• 85% reported a need for management training
	• Training preferences: during residency, interactive, by physician or extramural expert, topics: negotiation skills, specialist partnerships, the health care system, career opportunities and leadership
Competencies and needs of residents perceived by specialists [9]	• Inadequate perceptions on residents abilities: contract negotiating skills, knowledge of the healthcare system and specialist department.
	• 94% reported a need for management training among residents
	• Training preferences: during residency, interactive, by physician or extramural expert, topics: the health care system, time management, leadership, legal aspects of medical errors and communication.

We updated our literature search to see if new articles on management curricula had been published since 2010. We used the same search strategy as for the earlier literature review [8]. We found nine new articles describing a management program designed for medical residents [12-20]. In combination with our previous literature review a total of 32 articles described management training programs which focused on medical residents. Most of these curricula were developed based on previous literature and personal experience. Only five programs were based on a needs assessment, four among the residents themselves [21-24] and one among their supervisors [25]. There was no consensus regarding the content, the timing in the overall curriculum, the length of individual training sessions, or the total duration of management training programs. Of the 32 programs described, several courses had subjective evaluations, but only eight had objective pre- and/or posttests to evaluate the effect of the training (Table 2). None used in-depth interviews as a qualitative approach to evaluate the training courses.

**Table 2:** Overview pre-posttest designs in the literature

	**Number of items (design)**	**Reliability reported**	**Timing of posttest**	**Control group**
Crites	12 (true/false)	No	Immediately after completion	No
Babitch	5 (unknown)	No	Immediately after completion	No
Essex	65 (true/false)	No	Immediately and one month after completion	Yes
Hemmer	20 (unknown)	No	Before last session	No
Lopresti	40 (multiple choice and “pick N” questions)	Yes	Unknown	Yes
Turley	50 (multiple choice)	No	Unknown	No
Kerfoot	26 (multiple choice)	Yes	Immediately and five weeks after completion	Yes
David	10 (true/false)	No	Unknown	Yes

Based on the findings from our previous research, we decided to design and develop a management training program for medical residents. Our goal was 1) to develop a management course on the basis of a list of items suggested by residents and specialists, 2) to evaluate the objective and perceived impact of the course on the residents, and 3) to compare the knowledge of the healthcare system between residents and specialists.

## Methods

### Topic selection

Medical management is a broad subject and it is impossible to cover all areas in one training session. We therefore designed a training module and chose two themes from the list of suggested areas by the residents and specialists, namely knowledge of the healthcare system and time management. We chose these topics since we wanted to combine a theoretical topic (knowledge of the healthcare system) with a skill (time management). Also these topics were indicated as important in the literature review as well as in the needs assessments (Table 1).

### Instructors selection

To meet the preferences of the residents we wanted the instructors to be physicians as well as content experts. Members of the board of directors of the two participating hospitals (Atrium Medical Centre, Heerlen and Maastricht University Medical Centre, Maastricht) were approached to participate in the course as teachers. A member of the board of directors from each hospital offered to be an instructor on the topic “Knowledge of the healthcare system”. One of the divisional directors of the Atrium Medical Centre offered to be the instructor on “Time management”.

### Participants

In the two participating hospitals, residents in Paediatrics, Obstetrics and Gynaecology (ObGyn), Orthopaedic Surgery and Internal Medicine were approached for participation in the management training to obtain a representative group of residents from surgical- as well as non-surgical specialties. To implement an interactive learning approach, as was preferred in the needs assessments, we wanted no more than 15 participants in each group.

### Format of the training

The format of the course was set up to meet the requirements of the inter-disciplinary educational standards that apply in the south-east region of the Netherlands. The first session during week one, consisted of a two hours lecture on the concepts of the organization and financing of the healthcare system on national level and a two hours lecture on the general concepts of time management. Both sessions used a PowerPoint presentation as a general guideline to cover the most important subjects, but the instructors invited the participants to ask questions and bring their own thoughts on the subject to the table to create an interactive learning environment.

The residents received two homework assignments. With the first assignment they were invited to solve a simulated problem in the healthcare system and to present their solutions in a PowerPoint presentation. For the other assignment the residents received the documentation of a real staff meeting from one of the hospitals. They were asked to prepare themselves individually for the next session where a staff meeting would be simulated to teach them on time management and skills to effectively participate in and chair a staff meeting. Lectures and literature were provided to support the residents in their assignments.

After three weeks the second and final session of four hours took place. In the first hour the residents presented their solutions on the assigned problem in the healthcare system. Then another hour was spent on teaching them the micro concepts of the healthcare system, namely the organization and financing of hospitals and specialists departments. The session was concluded with a simulated staff meeting.

### Evaluation

#### Training course

Feedback from the residents was received through evaluation forms and in-depth interviews. The anonymous evaluation form consisted of five statements querying the residents on their assessment of each presentation. They were asked to rate the sessions on a 1–5 Likert scale (1 = very poor; 5 = very good) on the following points: the communication skills of the presenter, the extent to which the theoretical framework was clearly explained, the translation of theory into practice, the quality of the PowerPoint presentation and the overall usefulness of the presentation. In addition they were invited to rate the choice of the selected topics in the training, the quality of homework assignments, the provided literature, and the overall added value of the course. There was also an open area where they could leave their additional comments.

With the in-depth interviews we tried to gain further insight in the positive aspects of the training and the areas for improvement. A selection of residents (based on demographic information) and all three teachers were invited to further explain in a 20-minute telephone interview, their views on the management training. LB conducted the semi-structured interviews after the last training session. The interviews were guided by 12 questions covering the most important topics (strengths, points for improvement, topic selection, time per topic, format of the training, teachers, homework assignments and the literature). Participants were assured of anonymity and confidentiality and received the transcript of their interview by e-mail with the request to correct them if necessary.

#### Knowledge

To evaluate the effectiveness of the training a knowledge test consisting of 50 true/false-questions on the subject “knowledge of the health care system” was set up by the two instructors on that topic. Scores were expressed as the percentage correctly answered questions. The residents were also requested to complete an additional questionnaire to obtain information on background variables such as a resident’s work experience in years, if the resident attended management training before and if the resident had previous management experience.

### Procedures

#### Ethical approval

Prior to starting the (pilot) training we sought for ethical approval from the hospital’s research and ethics committee. They ruled that ethical approval was not required according to the Dutch Medical Research (Human Subjects) Act.

#### Pilot

We first conducted a pilot training from March till April 2012 at the Atrium Medical Centre. Eleven residents (6 from ObGyn, 1 from Internal Medicine and 4 from Orthopaedic Surgery) participated in the pilot training. Based on the analysis of the feedback (eleven evaluation forms and four in-depth interviews) we received about the pilot training, minor modifications were performed on the main format of the training workshop. For example, the homework assignment on the staff meeting was cancelled and the time schedule per subject was adjusted. The residents wanted more time (3 hours per session) on knowledge of the healthcare system and less on time-management and participating in or leading (staff)meetings (1 hour per session).

#### The definite training

**Figure 1:**
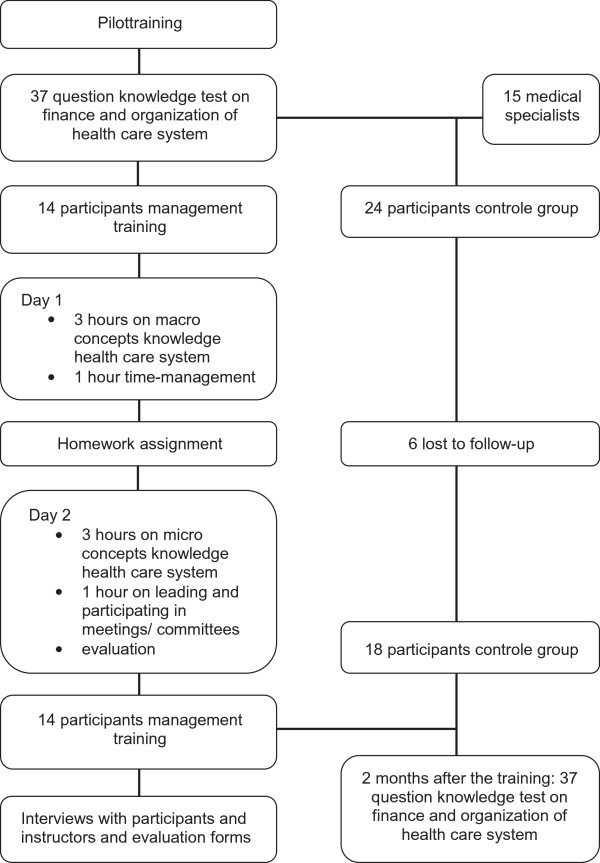
Flowchart pilot training.

The definite training ran from April till May 2012 at the Maastricht University Medical Centre. Again residents from Paediatrics, ObGyn, Orthopaedic Surgery and Internal Medicine were approached for participation. We also set up a group of medical residents from the same disciplines who served as a control for comparison. An email with information regarding the pilot training was sent to the respective residency directors, who forwarded the email to their residents. Interested residents could sign up for participation. The residency directors also provided the emailadresses of the non-participating residents. These residents were approached for participation in the control group. The assignment to experimental or control group was therefore largely determined by the residents’ availability and interest (i.e. convenience sampling). All program directors agreed to have their residents’ join in and facilitated participation as much as possible. Consent was sought for and obtained from all participating residents. Two weeks before the start of the training the participating residents and the residents in the control group were asked to fill in the 50 item questionnaire of knowledge testing (pre-test). At the same time medical specialists of Pediatrics, Orthopedics, Internal Medicine and ObGyn received the test to explore whether their knowledge of the healthcare system was gained over time through work experience. Immediately after the training, the participating residents filled in the evaluation forms. In the weeks after completion of the training, four in-depth interviews were performed among the participating residents and three among the teachers. After two months the knowledge test was again send by email to the participating residents and the residents in the control group (post-test) (see Figure 1).

### Data analysis

Analysis was performed using SPSS, version 17. Descriptive statistics were used to present the demographic distribution of the participants of this study. The reliability (Cronbach’s alpha) of the 50 true/false questions test was calculated using the pre-test data of all medical residents (intervention as well as control group). Whether the scores on the post-test were influenced by participating in the management course or not, was investigated using multiple regression analysis. The posttest score was the dependent variable in the analysis and training (0: control, 1: intervention), the pre-test score, and work experience were the independent variables. For ease of interpretation and numerical stability the centered version of the pre-test score, score pre-test – mean score pre-test, was used as independent variable in the regression analysis. Work experience was defined in years. We used an independent t-test to see if medical specialists had on average a significant higher score than the residents by comparing the pre-test results of all residents to the test results of the medical specialists. The in-depth interviews were transcribed verbatim. To classify these unstructured data into coding categories author LB created a coding dictionary by which all interviews were analyzed. Descriptive statistics were used to present these data.

## Results

### Final version of the knowledge test

The original test consisted of 50 true/false questions. In the reliability analysis, questions that did not positively contribute to the reliability were candidates to be removed. The coverage of the domain of the test was also taken into consideration when deciding on removing items. Using these indications 13 items were removed, resulting in a satisfactory alpha of .702 for the remaining set of 37 items in the final version of the test.

### Characteristics

Fourteen residents (2 from ObGyn, 5 from Internal Medicine, 5 from Orthopaedic Surgery and 2 from Paediatrics) participated in our final training. The control group consisted of 24 medical residents (3 Orthopaedic Surgery, 5 Paediatrics, 9 Internal Medicine, 7 ObGyn). The fourteen residents who participated in the course completed all sections of training and evaluation. Of the 24 residents in the control group six were lost to follow-up (3 ObGyn, 3 Orthopedic Surgery) and did not complete the post-test. There were no reasons given for not completing the post-test.

The group of medical specialists consisted of 6 Paediatricians, 5 Internists and 4 Gynaecologists. The participating residents had on average 5.6 years of work experience. The residents from the control group had on average 4.8 years of experience, while the medical specialists had an average of 20.3 years experience. Of the participating residents, one resident had previous management training while two residents had previous management experience. In the control group two residents had previous management experience and two residents had previous management training. Five of the 15 medical specialists had previous management training and 8 specialists had previous management experience. The residents in the intervention group scored on average 66.41% (SD 6.2) questions correct on the pre-test. The residents in the control group scored on average 67.94% (SD 9.7) correct. The specialists answered on average 71.23% (SD 8.1) of the questions correct. On the post-test the scores for the residents in the intervention and control groups were 72.97% (SD 5.7) and 71.22% (SD 5.2), respectively. Although the specialists had on average a higher score than the residents the difference, 4.06%, was not significant (p = 0.072) using independent T-test analysis (Table 3).

**Table 3:** Characteristics

	**Participating residents (n = 14)**	**Residents in control group (n = 24)**	**Medical specialists (n = 15)**
Specialisation			
• Orthopedical surgery	5	3	0
• Paediatrics	2	5	6
• Internal medicine	5	9	5
• Gynaecology	2	7	4
Work experience (years)	5.6	4.8	20.3
Previous training	N = 1, 7.1%	N = 2, 8.3%	N = 5, 33.3%
Previous experience	N = 2, 14.2%	N = 2, 8.3%	N = 8, 53.3%
Average score pretest	66.41% (SD 6.2)	67.94% (SD 9.7)	71.23% (SD 8.1)
Average score posttest	72.97% (SD 5.7)	71.22% (SD 5.2)	

### Multiple regression analysis

None of the independent variables had a significant influence on the post-scores. The score difference between intervention and control at post-intervention time was equal to 2.3% (p = 0.30; standard regression coefficient (src) = 0.21), the effect of the pre-test score on the post-test score was equal to 0.27 (p = 0.094; src = 0.33), and the effect of work experience was 0.11 (p = 0.82; src = 0.05).

### Evaluation forms

On a 1–5 Likert-scale (1 = very poor; 5 = very good) the items were assessed as follows:

● The average quality of the sessions: 4.21 (SD 0.45)

● The choice of the selected topics in this training: 4.09 (SD 0.30)

● The added value of the course: 4.27 (SD 0.65)

● The quality of the provided literature: 3.41 (SD 0.58)

● The homework assignments: 3.82 (SD 0.98)

The overall grade with which the residents rated the training was on average (on a scale from 1–10) a 7.66 (range 6–9). There were also some additional comments written on the evaluations forms. Two residents stated that they had too little basic knowledge of the subjects to fully appreciate the training. Two residents stated that this training filled a gap in their current postgraduate medical training. Three residents thought that the homework assignments were too broad, they advised to limit the assignments so that the presentations of the assignments wouldn’t take up as much time during the sessions. Five residents wrote that they would have liked even more opportunities for debate during the sessions.

### In-depth interviews

Four residents (1 ObGyn, 1 pediatrics, 1 orthopedics, 1 internal medicine) and the three instructors participated in the in-depth interviews.

#### Residents

All four residents stated that they had appreciated the training, some positive points they named were:

● *“The teachers used an interactive approach.”* (resident 1)

● *“It fills a gap in our current postgraduate medical training.”* (resident 2)

● *“I would like to have another session, my interest is piqued.”* (resident 3)

There were also some points for improvement. Three out of four residents said that too much basic knowledge was expected as resident 1 stated: *“I had heard of some basic concepts and perhaps I should have asked more but occasionally it really was like Chinese to me.”* Also three out of four residents said they liked the fact that they had received some literature before the start of the training, but two out of those three said that the amount had been too much and that they rather would have received two short overview articles.

All four residents appreciated the topics that were chosen, although resident 4 said that he would maybe split the topics up. *“The topics are well chosen, but they do not fit in the same course. Time management is more suitable for first year medical residents and the organization and financing of health care is better appreciated by senior residents”.* We also asked them if they had missed a topic in this training course. Two out of four said that they would like to have heard more about the organization and financing of specialist partnerships.

They all liked the format of the training and they would preserve the two sessions as *“it is too much information for one day.”* (resident 1 and 4). They also appreciated the homework assignment in which they had to solve a simulated problem in the healthcare system and present their solutions, *“it is a trigger for some interesting discussions”* (resident 4), and *“a homework assignment serves as a big stick to ensure that you delve into the material” (resident 1)*.

There was some discussion on the question if they had liked the fact that all teachers were also physicians. Two out of three said yes, because “*they were better able to empathize with the bustle of the day and estimate what material was interesting for us*.” One said yes on time-management, but he didn’t think it was of added value on the topic organization and financing of the health care system. And one said no, “*because their stories sometimes seemed a bit biased*.”

#### Teachers

All three teachers agreed on the topics that were being taught. An additional comment was: “*There are many other important issues to consider, but in the limited time we had these topics were adequate and relevant”* (teacher 2).

If they had to pick another topic, two out of three said that they thought that the residents probably wanted to know more about specialist partnerships.

They all wanted to keep the current format, *“it is too much information for one day*” (teacher 3) and teacher 2 stated that “*this format encouraged residents to get involved with the material”.*

The two teachers on the subject “knowledge of the organization and financing of the health care system” stated that they had expected a higher level of basic knowledge from the participating residents.

## Discussion

In this study, we set out to design and develop a management training module for medical residents. We also wanted to evaluate the objective and subjective impact of the course on the residents, and to compare the objective knowledge of the healthcare system between residents and specialists.

We developed a training course in which two management topics were taught, namely “the organization and financing of the health care system in the Netherlands” and “time management”. We used a pre- and post- knowledge test to see if the knowledge of the participating residents on the “organization and financing of the health care system” had significantly improved after participating in the training in comparison to the residents in the control group. Although the difference between the percentages of correctly answered questions on the pre- and post-test was greater for the participating residents (6.56%) in comparison to the residents from the control group (3.28%), the difference was not significant. This can be explained by four possible causes. First of all our research groups were small and probably too small to detect a significant difference. To detect a large effect with a power of 80% and a significance level of 5% at least 26 persons had to take part in the participating group as well as in the control group. Due to a deadline for this project, we weren’t able to provide those numbers. Secondly, the knowledge test was based on the Powerpoint presentations the teachers had prepared, but due to a lack of basic knowledge of the residents on the topics and due to the vivid discussions (which were encouraged), the teachers did not complete their Powerpoint presentations. Although the residents received the Powerpoint presentations by email, it is possible that they did not pick up on all information that was available. Also, by using true/false questions residents who didn’t know the answer to a question still had a 50% chance of getting it right causing the final scores to be generally higher. This could have added to the difficulty of reaching significant differences in the pre-and post-tests. Finally it is also possible that the knowledge the residents gained from this training was not retained as well as we had hoped for.

Of the eight management training programs described in the literature, which also used objective pre- and posttests, four programs found a significant increase in knowledge [13,14,25,26]. Three out of those four programs had a research population of less than 35 participants [14,25,26]. The other four programs described an increase in knowledge, but this increase was either not significant, or not tested for significance.

Another possible limitation in our study is the number of participants lost to follow-up in our control group. Of the tweny-four residents in the control group, six were lost to follow-up, while none were lost to follow-up in the intervention group. A possible cause for this difference could be due to a different level of commitment and interest between the groups. For example, the residents from the intervention group received a homework assignment and training while the control group only had to fill in the questionnaire twice. It is therefore possible that the least interested residents from the control group, dropped out first. However, these drop-outs may have resulted in the two groups being better comparable, for which a negative influence on the reliability of our results is not expected.

The 15 medical specialists did not significantly score better on the knowledge test than the residents. Even though the specialists had relatively more work experience, management experience and previous management training in comparison to the residents. This could suggest that work experience alone is not enough to gain knowledge on this subject. But the group of participating specialists and residents was again too small to give a definite answer to this question or to perform sub analyses on the variables management experience and management training.

The evaluation of the participants’ perceptions showed that all residents appreciated the training. Some of them stated that it had filled a gap in their current specialist training and others said that they would have liked even more education on these subjects. Residents and teachers agreed on the topics that were chosen for this course, but some suggested that specific knowledge on how specialist partnerships in the Netherlands are organized and financed would also be of value, especially to those who are almost at the end of their specialist training. This evaluation suggests that two sessions of four hours isn’t enough to adequately teach all aspects of medical management and to satisfy the residents educational needs on this matter. Besides “specialist partnerships” the needs assessment and literature review we earlier performed showed that topics as negotiation skills, career opportunities, legal issues and leadership skills are also deemed important [8,9,11]. At least two other training modules would be necessary to teach all these subjects. Our suggestions would be to divide the topics into subjects suitable for junior medical residents and for residents who are more advanced in their specialist training program. In the literature, of the 32 training programs described, 21 used a subjective evaluation of which 21 [14,15,17,19,20,22,27-40] were positive and one was neutral [13]. Based on our own evaluation and the evaluations in the literature we also suggest that medical management training should be mandatory since we feel that all doctors should have a basic knowledge of these subjects and residents seem to have a need for it.

For future research in this area, we suggest that larger intervention and control groups should be used to correct for eventual fallouts and increase the reliability of the findings. In addition it would be interesting to investigate if not only knowledge on these topics is maintained but also to examine if this training leads to improvements in daily practice. In particulair with regards to their time management skills.

## Conclusion

We recognize that most postgraduate medical curricula are already quite full and that there is little room left for additional content, namely medical management. However we think that since the managers role has been identified as a key competency in the Netherlands but also in many other countries (US, Canada, Australië), the training programs in those countries need to design courses to develop this competency. This paper has described how we developed and evaluated a management training module, which taught the topics “organization and financing of the health care system” and “time management”. This training was evaluated positively and considered to be of added value by the participants. This training is an example of how to systematically develop, design and evaluate management training courses for medical residents. Based on this and our previous research experience in this area, we recommend that medical management training should be a mandatory part of the postgraduate medical curriculum.

## Competing interests

The authors declare that they have no competing interests.

## Authors’ contributions

LB has made substantial contributions to conception and design, acquisition of data, analysis and interpretation of data. LB also has been involved in drafting the manuscript and gave final approval of the version to be published. AM has made substantial contributions to analysis and interpretation of data. AM also revised the manuscript critically for important intellectual content and gave final approval of the version to be published. LZ has made substantial contributions to conception and design, he has made substantial contributions to acquisition of data. LZ also revised the manuscript critically for. IH has made substantial contributions to conception and design, he has made substantial contributions to acquisition of data. IH also revised the manuscript critically for important intellectual content and gave final approval of the version to be published. AS has made substantial contributions to conception and design. AS also revised the manuscript critically for important intellectual content and gave final approval of the version to be published. JB has made substantial contributions to conception and design and the acquisition of data. JB also has been involved in drafting the manuscript, revised the manuscript critically for important intellectual content and gave final approval of the version to be published.

## Pre-publication history

The pre-publication history for this paper can be accessed here:

http://www.biomedcentral.com/1472-6920/14/107/prepub
